# S202. EFFICACY OF LONG-TERM RESIDENTIAL TREATMENT FOR PERSISTENT MENTAL ILLNESS

**DOI:** 10.1093/schbul/sby018.989

**Published:** 2018-04-01

**Authors:** Michael Knable

**Affiliations:** Sylvan C. Herman Foundation

## Abstract

**Background:**

In the United States, the number of public and private psychiatric hospital beds has steadily declined in recent years, despite the lack of intensive intermediate care alternatives in the community. The design and implementation of intensive residential treatment programs are not currently guided by controlled studies, but these studies are necessary to determine the clinical and economic utility of such programs. We present clinical and outcome data on an initial sample of patients treated over the last 5 years.

**Methods:**

Naturalistic, non-controlled assessment of symptomatic and functional outcome in an initial sample of young adults with persistent mental illnesses treated in a community-based residential program. Patients were treated with an individualized combination of modalities such as Illness Education and Management, Supported Employment, Individual, Group and Family Psychotherapies and Psychopharmacology. Standard clinical rating scales were used during the period of treatment and all discharged patients were contacted on an annual basis in order to complete a survey of clinical outcome.

**Results:**

101 patients had been admitted and treated since the facility opened in October 2011. Median age of the patients was 25 years, mean illness duration was 12.6 years, and the mean number of prior hospitalizations was 6.5. Diagnostic distribution was: 36.7% psychotic disorders, 27.7% unipolar mood disorders, 19.8% bipolar mood disorders, 7.9% autism spectrum disorders, and 7.9% post-traumatic stress disorder or other anxiety conditions. 37% of residents met criteria for personality disorders, the majority of which was borderline personality disorder. 42% of residents also met criteria for a substance use disorder in the year prior to admission. Ratings on the Multnomah Community Ability Scale improved by 16%, ratings on the Brief Psychiatric Rating Scale declined by 20% and ratings on the Montgomery-Asberg Depression Rating Scale declined by 37%. The average survey response rate after discharge was 59%. With regard to community engagement: 40.3% of current residents and 35.1% of discharged residents were competitively employed. 16.7% of current residents and 17.8% of discharged residents worked as volunteers, and 23.3% of current residents and 26.3% of discharged residents were attending school. A survey of dispositions revealed that: 49.7% of discharged residents were living independently, 14.9% were living with family, 2.2% were homeless, and 5.4% had died from suicide. The hospitalization rate declined from 0.84/year to 0.57/year before and after discharge.

**Discussion:**

Long-term residential treatment for young adults with persistent mental illness results in improved symptomatic recovery, independent living, increased employment rates, and reduced hospitalizations.

